# Group weight management program for people with schizophrenia or bipolar disorder: an Italian experience

**DOI:** 10.1192/j.eurpsy.2025.2232

**Published:** 2025-08-26

**Authors:** V. Martiadis, F. Raffone, E. Pessina, E. Favaretto, A. Martini, P. Matera, C. I. Cattaneo

**Affiliations:** 1 Department of Mental Health, Asl Napoli 1 Centro, Naples; 2 Department of Mental Health, Asl Cuneo 2, Bra; 3 Department of Addiction, South Tyrol Health Care, Bressanone; 4 Department of Mental Health, Asl Biella, Biella, Italy

## Abstract

**Introduction:**

Obesity and weight gain are major clinical problems for people with severe mental illness (SMI), such as schizophrenia and bipolar disorder. While psychopharmacological treatment, particularly with atypical antipsychotics and mood stabilisers, is the ‘core’ of treatment for these disorders, it can increase the risk of overweight/obesity, metabolic and cardiovascular disease. Most guidelines on how to manage overweight and obesity in these patients share an initial conservative approach. These guidelines include diet, physical activity, lifestyle coaching and behaviour modification. Only then are pharmacological or, eventually, surgical treatments added.

**Objectives:**

This study evaluated the effectiveness of a group behavioural weight management program in a real Italian outpatient setting.

**Methods:**

100 patients diagnosed with schizophrenia or bipolar disorder who participated in a group weight management program were followed up for 12 months. The intervention consisted of 8-week training in which patients received nutritional and lifestyle coaching in groups of 10. Weight, BMI, waist circumference, blood glucose and blood pressure were measured at 0, 6 and 12 months.

**Results:**

Mean body weight (kg) decreased from 98.01±18.30 at baseline to 93.29±17.36 at 6 months (p>0.001) and to 90.35±17.90 at 12 months. There were also statistically significant reductions in BMI, waist circumference, blood glucose and systolic blood pressure. There was no significant reduction in diastolic blood pressure. After segmenting the patients at baseline according to their initial body weight (normal weight, overweight and obese according to the World Health Organisation), a statistically significant difference in weight only occurred between baseline and the first 6 months of follow-up, suggesting that the programme was successful in the short term and that the results were maintained over the following 6 months.

**Conclusions:**

Despite the study’s limitations, the intervention demonstrated feasibility in an outpatient setting, a high retention rate with no drop-outs during the programme, and significant weight loss in the first six months, followed by long-term maintenance at the end of the study. Current NICE recommendations suggest that people with SMI, particularly those receiving antipsychotic treatment, should receive integrated diet and exercise programmes. Future research should focus on the cost-effectiveness of this type of intervention and its reliability in the medium and long-term in different health care settings.

**Disclosure of Interest:**

None Declared

